# Myeloma patients’ experiences of a supervised physical activity programme: a qualitative study

**DOI:** 10.1007/s00520-022-07062-x

**Published:** 2022-04-25

**Authors:** Joanne Land, Julia Hackett, Govundeep Sidhu, Malgorzata Heinrich, Orla McCourt, Kwee L. Yong, Abi Fisher, Rebecca J. Beeken

**Affiliations:** 1 Department of Behavioural Science and Health, University College London, 1-19 Torrington Place, London, WC1E 7HB UK; 2 Cancer Institute, University College London, London, UK; 3 Martin House Research Centre, University of York, York, UK; 4 Leeds Institute of Health Sciences, University of Leeds, Leeds, UK

**Keywords:** Cancer, Cancer survivors, Exercise, Multiple myeloma, Oncology, Physical activity, Qualitative research, Quality of life, Rehabilitation

## Abstract

**Purpose:**

The Myeloma: Advancing Survival Cancer Outcomes Trial (MASCOT) tested the impact of a supervised exercise programme on fatigue, clinical, and patient-reported outcomes in multiple myeloma [MM] patients. The current study explored MM patients’ experiences of the programme to guide future interventions.

**Methods:**

Purposive sampling was used to recruit stable MM patients participating in MASCOT. Semi-structured, face-to-face interviews were conducted, transcribed verbatim, and analysed using thematic analysis.

**Results:**

Six themes were identified. Key drivers for participation in MASCOT were “Altruism and extended cancer care”; participants wanted to give something back and assist in improving post-treatment care for MM patients, especially as after treatment “Barriers to being physically active” were a fear of damage and lack of health professional guidance. “Influences fostering change within the intervention” included physiotherapy supervision and tailored exercises, which gave participants confidence to push themselves in a safe environment and broke down misconceptions about their body. “Social support”, from both family and peers in the programme, promoted motivation and adherence. Participants expressed concerns about “Maintaining things going forward” but had identified mechanisms to aid continuation. “Physical and mental benefits” of the programme were highlighted; participants were able to do things they couldn’t before and described feeling free from the constraints of MM.

**Conclusions:**

A post-treatment exercise intervention for MM patients was a positive experience, which enhanced participants’ physical and psychological wellbeing. Tailored gym and home-based exercises, a specialist cancer physiotherapist, and sustained support were perceived to be important for success.

**Implications for cancer survivors:**

Exercise support for MM patients, ideally with physiotherapist supervision, should be incorporated into survivorship care to qualitatively improve patients’ quality of life, self-efficacy, and mental wellbeing.

**Supplementary Information:**

The online version contains supplementary material available at 10.1007/s00520-022-07062-x.

## Background

Myeloma is a malignancy of the plasma cells in the bone marrow. It affects 9.6 per 100,000 individuals per year in the UK and incidence rates are projected to rise to 16 cases per 100,000 by 2035 [1, 2]. MM is incurable and patients undergo intensive treatment followed by periods of remission before a relapse occurs and they require further treatment. With the introduction of novel treatments and autologous stem cell transplantation (ASCT), patients’ 5- and 10-year survival has increased to 50% and 29% respectively in 2018 compared to 37% surviving 5 years between 2005 and 2009 [3]. However, during treatment-free periods, MM patients report a high symptom burden, which negatively affects their quality of life (QoL) and reduced their ability to participate in social and physical activities [4].

Exercise interventions have the potential to mitigate some of these effects [5]. A systematic review of 18 exercise studies in haematological cancers (including myeloma) found exercise may reduce fatigue [6]. In myeloma specifically, 2 pilot studies of exercise found post-intervention improvements in QoL, fatigue, and strength [7] 8, but a randomised controlled trial (RCT) of an exercise intervention versus usual care for 187 MM patients receiving treatment failed to show any significant differences in outcomes between groups [9].

The Myeloma: Advancing Survival Outcomes Trial (MASCOT) was the first RCT to investigate the effects of a physiotherapist-led exercise programme for MM patients who have completed treatment. Results showed improvements in leg strength and a trend to improving aerobic fitness for those who exercised, but no differences in fatigue or other patient-reported outcomes at 3 or 6 months between the groups [10]. However, unlike the general myeloma population [4], this sample had low levels of fatigue at baseline, were functionally ‘well’ (ECOG scores mainly 0), and reported high baseline levels of wellbeing and QoL [10]. In addition, although standardized patient-reported outcomes were used, these are not always sufficient to capture patients’ perspectives of the meaningfulness of exercise related to post-treatment recovery. Qualitative data are required to unpick these issues.

Coon and Coleman [11, 12] have previously explored myeloma patients’ experiences of supervised exercise using qualitative methods in the USA. Twenty-one patients taking part in a home-based exercise programme were interviewed about their experiences of an intervention for fatigue [13] and facilitators and barriers to engagement [14]. Overall, participants believed exercise would be good for them; participating made them feel they were personally doing something positive to support their own treatment and reduce their fatigue. This belief helped facilitate their exercise journey along with support from family and health professionals. Barriers to exercise were symptoms of treatment and their environment i.e. weather or schedule demands [11, 12].

These exercise barriers were also identified by Craike et al. (2013), who interviewed 24 MM patients in Australia. Patients were prospectively asked what their preferences would be for an exercise intervention to overcome these barriers, and individualised exercises, ability to socialise with others with MM, and a myeloma specialist to run the class were suggested. There was 50/50 support for a home-based programme and supervised hospital-based programme [15]. The MASCOT study drew on this data and utilised these components within the tested intervention. Understanding participants’ experiences of an exercise programme that combines home-based exercises with a supervised programme in a hospital gym will help to further the knowledge within this field and inform interventions for this group going forward [10].

Therefore, the aim of this study was to (i) examine MASCOT participants’ experiences of following a supervised gym-based exercise programme and (ii) explore aspects of the programme that worked well or could be improved to aid future exercise interventions for this patient group.

## Methods

### Participants and procedure

The first 30 participants who completed the exercise intervention for the MASCOT study were approached to participate in the qualitative study [10]. Inclusion and exclusion criteria for the MASCOT study can be found in Table 1. Participants were recruited to MASCOT from a myeloma outpatient clinic at a London hospital. The MASCOT exercise programme was delivered by a physiotherapist (JL, OM) in the hospital gym and included a mix of aerobic and strengthening exercises. For the first 3 months, patients were supervised once per week in the hospital and expected to exercise twice per week at home. In the following three months, they exercised at home three times per week.

**Table 1:** Inclusion and exclusion criteria

Inclusion criteria
Stable disease for at least 6 weeks, completed their initial treatment, or were on maintenance therapy
ECOG performance status 0–2
Able to undergo a regular exercise programme
Exclusion criteria
Spinal instability (as assessed on radiology in multi-disciplinary team meetings)
Recent (within 4 weeks) spinal or other surgery for pathological fractures
Abnormal resting electrocardiogram, where clinically indicated unexplained by further cardiological work-up
At risk of pathological fracture based on Mirel’s score
Current participation in an exercise programme as part of a research study
Unstable angina
Musculoskeletal disease limiting mobility
Cognitive impairment that impedes ability to complete questionnaires

Participants received a logbook to record exercises and a booklet containing goal setting tools based on habit theory intended to support behaviour change (details in supplementary material) [13]. Full details can be found in the trial paper [10].

Ethical approval for MASCOT (including the qualitative interviews) was provided by Queens Square Ethics Committee (13/LO/1105) and the trial is registered (ISRCTN 38,480,455). All participants provided informed written consent prior to enrolling.

### Data collection

Between April 2015 to November 2016, twenty face-to-face interviews were conducted with consenting participants in a private room at the hospital research centre. Interviews were conducted during participants’ 6-month assessment, once they had completed the supervised intervention. The interviews were conducted by two female behavioural scientists (RJB & SP) with experience interviewing cancer patients. RJB was part of the team who conceptualised the study and wrote the interview guide, but neither researcher was involved in the delivery or administration of the intervention. Participants had not met the interviewers prior to the interview. A topic guide explored patients’ motivations for participating in the trial, general trial feedback, and how they found the exercise intervention and behaviour change support (supplementary file). Interviews were audio-recorded, transcribed verbatim, and anonymised. Half (50%) of the recordings were checked by JL against the interview transcripts to verify accuracy. No repeat interviews were carried out.

### Data analysis

Descriptive statistics were used to summarise the interview participants’ physical activity data and demographic and clinical characteristics.

NVivo 12 supported a thematic approach to analysis. Ten percent of the transcripts were double coded by (JH and JL) for inter-coder reliability, and a coding schedule was developed. JH/GS coded the remainder of the transcripts. Data were initially coded deductively to areas pre-specified in the topic guide; further codes were identified inductively. Interview transcripts were coded using the method of constant comparison, whereby data are compared systematically for similarities and differences and coded accordingly, with discrepant accounts being sought. Codes were grouped to form overarching themes, which were iteratively refined over the course of the analysis [14]. The COREQ Checklist is within the supplementary information.

## Results

Of 30 MM patients approached, 2 withdrew (no reason given), 2 were medically excluded (kidney transplant, myeloma not stable), and 6 did not respond to a verbal reminder. In total, 20 interviews were completed. The majority of participants were White (90%), were male (70%), and ranged from 48 to 78 years of age, with a median age of 64. Table 2 displays participants’ characteristics and Table 3 their clinical characteristics. The mean average interview length was 40.14 min SD 12.12 (range 16.16–62). The majority (95%; 19/20) of interviewees reported they would recommend the exercise programme to others and felt they derived psychological and/or physical benefit from taking part. One of the recordings was cut short due to a technical error and therefore it is unknown if this patient went on to recommend the programme.

**Table 2:** Demographic characteristics of the study population

Demographic	Participants (*n* = 20)
Sex, *n* (%)	
Female	6 (30)
Male	14 (70)
Age	
Mean	64.75
Median	64
SD	8.71
Range	48–78
Ethnicity, *n* (%)	
White	18 (90)
Black	1 (5)
Other	1 (5)
Living arrangements, *n* (%)	
Owner occupied	17 (85)
Council rented	1 (5)
Privately rented	2 (10)

**Table 3:** Clinical characteristics of the study population

Clinical	Participants (*n* = 20)
Myeloma isotype, *n* (%)	
IgA	4 (20)
IgG	11 (55)
Light chain	4 (20)
Non-secretory/oligo-secretory	1 (5)
Time since treatment in months	
Mean	26.9
Median	12
SD	25.48
Range	4–84
Surgery, *n* (%)	
Yes	3 (15)
No	17 (85)
Stem cell transplant, *n* (%)	
Yes	18 (90)
No	2 (10)
Meeting recommended exercise guidelines 150 min per week (%)	
Meeting guidelines	11 (55)
Not meeting guidelines	9 (45)
Disease status, *n* (%)	
Complete remission	5 (25)
Very good partial response	2(10)
Partial response	12 (60)
Stable disease	1 (5)
ECOG	
0	17 (85)
1	3 (15)

Six overarching themes (and 13 subthemes) were identified. These are summarised in Table 4 along with supporting participant quotes.

**Table 4:** Themes and subthemes

Main themes		Supporting quotes
Themes	Subthemes	
1—Altruism and perception of extended cancer care—key drivers for participation	- Altruism	*I felt that I’d had such good treatment with the transplant that I was giving something back. (Female, 77)*
	- Extended cancer care	*I took part because it could be good for me, and, people like me in the future. So as far as I'm concerned, the whole package could be made better for them… I just thought after treatment I could do nothing more other than just sort of sit about on the sofa. (Male, 64)* *You’ve been through all the treatments and all the stem cell transplants and all that stuff, you get very lethargic, and this was a very structured way for me to get out of that lethargy. (Male, 68)*
2—Barriers to being physically active after treatment	- Fear of damage	*I’ve always been a fairly fit person until I got myeloma, I used to belong to a running club…My wife says I’m not gonna run again and she’s the one that pulls the strings. She thought it might be dangerous for me, subsequently I gave up running. I wanted to run again, if I get the approval I might do a bit of jogging. (Male, 73)* *I was frightened to exercise before and again, this is a common theme I’ve heard from – from other people, but you know, for me I was frightened that I was gonna do some damage because you know, I’d done so much damage to my bones. I know that they’ve been impacted significantly. I’ve been through a lot of pain before through breaking it or… So – and I – and I’d had advice before which was highly cautious, so all of that combined together had made me very nervous about, you know, using my body and pushing myself. (Female, 48)*
	- Lack of health care professional guidance for exercise in usual care	*I er was referred to the […] Hospital […] and the advice I was given was, you know, really cautious. […] It was more about all the things you couldn’t do and the things I wasn’t allowed to do – you know. (Female, 48)* *I was always afraid of bending or lifting anything heavy and er I didn’t understand [..], myeloma, it’s upset my back. My bones have been damaged, you know. Like they explained. (Male, 56)* *there is an effort obviously somewhere um to learn about exercise and hopefully, to apply it, but on the other side, I see that there are no spaces in hospitals even for gyms, so I’m learning there is a contradiction. (Female, 77)* *I saw my consultant in the local hospital and told him what was happening [taking part in exercise programme] and he was sceptical thinking well, you’re healthy already, but I think he’s missed the point of it, I think you need to talk about the impact on them, how they’ve changed. It’s changed me, it has, in a positive way. (Male, 64)* *Nowadays somebody has a hip done and they get them up the next day and they’re doing exercise which is proved to be you know much better for them, perhaps in my treatment, in the handbook for myeloma incorporating gentle exercise that increases could well become part of it because it’s so easy to become bed bound, house bound, whatever – and it’s an illness that the mental side is often the dangerous side. (Male, 64)*
3—Influences fostering change within the exercise class	- Importance of physiotherapist supervision	*The confidence of having a really highly skilled physio supporting me physically and kind of mentally and emotionally through that was brilliant. It really gave me the confidence and ability […] to go out and have a more active life. (Female, 48)* *[The physiotherapist] was absolutely able to push me much harder than I would have pushed myself and gave me the confidence to know it was safe to do so, you know, so she really knew how to help me manage things in a safe way. (Female, 55)* *[The physiotherapist] has been my primary interface. Um, I can only, […] compliment her […] she’s been the motivation for me to do this to a certain extent. (Male, 68)* *I knew [the physiotherapist] was always at the end of the email, erm, and she was really supportive, […] she was, in my opinion, excellent. (Female, 77)* *I didn’t have anybody to ring up. You know, that was the other thing. I’ve just suddenly thought of that. It might have helped if there was a bit […] the participants were encouraged, […] I really did feel that I couldn’t ring [the physio] because I thought she’s probably rushed off her feet, really busy. (Female, 64)*
	- Tailored exercise	*Yeah. I mean, it’s really good because the first week, [the physio] wanted me to, you know, bend my knee, do a sort of a squat and I said, ‘I can’t do that. My knees are bad. I can’t bend them like that.’ And she said, ‘Oh, okay.’ So she just altered the exercise a bit. […] now I can do squats so I’m nearly sitting down. (Female, 60)* *I’m really glad I did do it because I think the danger is you decide “oh well, that’s the end of my life, I’ll just fade away”, you know, so you really need some encouragement to do some of the things which you would normally do. Now I’ve got a good idea of what I can do and what I can’t do physically which is really rewarding because I had no confidence at the end of the chemo and most of that lack of confidence has more or less gone now. So just doing these 12 weeks to me has been a really good thing…I feel much more able to do things which I probably wouldn’t have done. (Male, 75)*
	- Limited engagement with behaviour change techniques	*I thought it would be a useful tool if it applied to you, […] I do believe there are plenty of people who get a condition like myeloma and they go, "Oh, I'm really ill; I can't do anything." And I think in that circumstance it's helpful. (Male, 48)* *Yeah. I mean, I’m, I’m afraid that, you know, setting goals and rewarding yourself, I’m probably a bit long in the tooth for that stuff now, you know. Um, I understand the, the reason for it, um, but I’ve achieved my own goals much better than, you know, writing it in a book. (Male, 68)* *It’s very helpful to have the book because it reminds you. There’s no escape really, you’ve got the book. (Male, 75)* *..the log book’s great because there’s a record, you know, which did help me. What it did sort of help me do was get more regular with the um – with the exercise and I think having that written record of what you were doing helped me. It, you know, helped me stay on track so that was good, yeah. (Female, 48)*
4—Social support for exercise	- Social networks	*They encourage me, even my kids. Sometimes when I found myself being lazy, I got a push…They told me ‘Let’s go for a walk’, or running. So I always got the support. (Male, 64)* *Just- not the physical part because all over was working, but it’s the mental part of it. It’s the biggest benefit part. Because as I say, if I was going shopping and the wife says to me, ‘Don’t lift that. I’ll carry that,’ I used to let her do it. You feel embarrassed, you know. But now, I say, ‘No, no, no. I can do that.’ I can lift now [laughter] So that’s the key to it. That was the-mental part that you’re not just a cabbage. (Male, 58)*
	- Group exercise sessions	*I saw some patients that were not as lucky as I had been. Some patients that had had real problems to their backs, for example. There was a person there who had a steel rod in his back and it was great to see how he was still exercising, still doing things, and that was good for me to see because you think, ‘Oh well, if that happens to me one day, it means I can still do those things. I can still carry on in a relatively normal way’. (Female, 56)* *It was nice in terms of as a side effect I suppose, to speak to other myeloma patients’cause it’s not something you generally you know…I don’t belong to any support groups, so it was quite nice. You see familiar faces and it was nice to see them progress as well so there was a sense of camaraderie. It was nice. It was a very supportive environment. We’d be teasing each other a little bit, ‘Come on, you can do it,’ or, ‘Come on. Push a bit more.’ There was a nice environment. I think it was supportive. (Female, 48)* *The environment seemed to be quite appropriate to me, so that was fine, whereas in another environment I found it irritating to be with all of these people who didn’t want to talk about anything but bloody cancer all the time. But sharing one’s experience of physical exercise seemed to be a much more, much more positive thing to do actually. (Male, 75)* *I’m quite private about my illness and everything to do with it, […] I tend not to engage in conversation with other patients anywhere. (Female, 55)*
5—Maintaining things going forward	- Unsupervised exercise was challenging	*The exercises at home aren't as intensive. (Male 64)* *(if) there’s nobody with you, nobody pushing you… then you can get a bit lazy if you are not careful. (Male, 70)* *the problem now is, now that I’m only up there once a month, um the actual motivation […] I’ve been a bit tired recently and you just think, oh, that is the last thing I wanna do is exercises. (Female, 60)*
	- Mechanisms to continue exercise	*After I had been diagnosed, before I started in fact this programme, I wasn’t sure what I could and what I couldn’t do. I was afraid of pushing myself. I was afraid that I would cause an injury, that I would tear a muscle. I did not know what I could do. And now I know that I can do anything. That it’s not a matter of myeloma; it’s a matter of it hurts because it hurts. Um it hurts because it’s fatigued but it not because it’s myeloma. Now I can exercise and do whatever I do with peace of mind. It’s good. (Female, 56)* *It’s given me the motivation to carry on with at-home exercises…coming here every Tuesday is fairly regimented, but I do the exercises at exactly the same time each week now, so it’s taught me that. My wife and I will go round a lake near where we live, four and a half miles every Saturday morning. This has instilled in me to do that as a routine. I do, you know, TheraBand exercises at home at a specific time. Because I’ve got the discipline of coming here every week, or have had, and now I come here every week, I feel in myself I can carry that discipline forward to make sure I go to a gym. (Male, 68)* *At the moment you see someone once a week, don’t you, and you go through a similar set of exercises and that’s, that’s the support you need, but asking the NHS to give you a personal trainer is not going to go very far, is it? I don’t know how you’d do this but if it [drop-in session] was once a month, maybe that’s much more practical, and that might be enough actually, you never know. But I do think you need something other than “go away and do it on your own”. People vary, I know, but you do need some encouragement occasionally. (Male, 75)*
6—Physical and mental benefits	- Improved physical health	*Before I started all this, there was no way I could be walking up without touching the banisters." You know, it was a matter of holding on the banister and doing, one step and then the next step carefully, and now I can just sort of walk up and down the stairs without touching banisters and if it hadn't been for all these exercises that wouldn't be the case. (Male, 64)* *Yes, I can lift things. I don’t mean- don’t lift things crazy but I can lift things where I wasn’t before, so that knowledge really really helped me, you know, a lot and the physical part is brilliant because now I know I’ve got to do- I can do exercise. (Male, 58)* *… I really do feel very good. It’s amazing. I’m sure if I hadn’t have done these exercises, I mean, for instance, I, well, I live in a flat and, on the fourth floor and I couldn’t walk up the stairs to the flat but I go up and down two or three times a day now. (Female, 77)* *When I started, in my first assessment, I was pushing thirty-five kilos with one leg; by the end of the three months, I was pushing seventy kilos. (Female, 56)*
	- Improved self-efficacy	*I think the biggest thing has been that even though your body gets ravaged by myeloma and by the treatment, you can absolutely get back to an active, healthy lifestyle, that it’s possible. And I didn’t, I wasn’t absolutely sure that was possible and I hadn’t been given that confidence by anybody before this, in the whole process I’d gone through. (Female, 48)* *Well, the benefits is that I'm, you know, more like a normal human again, with standard things of life like walking and standing up and sitting down, that sort of thing. You know, because I can strap hang in the tube now, and things. (Male, 64)* *I do think that the spin offs are that exercise perhaps is earlier in the treatment for myeloma patients. (Male, 64)*

## Theme 1: Altruism and perception of extended cancer care—key drivers for participation

### Altruism

The majority of participants identified altruism as the driving force for participation in the study. They shared the belief that it was their way of giving something back to the medical system that had helped them.

### Extended cancer care

Participants reported that by taking part they hoped it would benefit future MM patients because they did not feel the care they received after treatment was sufficient for helping them return to pre-diagnosis activity levels. This was further shown by the majority of interviewees reporting they took part as a way to extend their cancer care. They felt deconditioned after treatment and this was a path to get fit.

## Theme 2: Barriers to being physically active after treatment

### Fear of damage

Prior to diagnosis, MM interviewees described themselves as being physically active or identified themselves as exercisers. They described how diagnosis and treatment had therefore had a big impact on their sense of self and identity. They became afraid to push themselves physically and refrained from doing activities which were previously routine. Their families often fed into this by dissuading them to do exercise, as they perceived this to be helpful.

### Lack of health care professional guidance for exercise in usual care

Participants felt discouraged that consultations with clinicians tended to focus on activities they should avoid, with some participants receiving advice to stop their usual hobbies because of their “damaged” bones. As a result of these discussions and language used, participants became fearful and stopped social activities and some even gave up work. Participants felt that there was no advice about what they could do and this was reinforced by a lack of encouragement to be physically active from health care professionals. Participants suggested that a leaflet or a discussion around their ability to participate in exercise would be beneficial.

## Theme 3: Influences fostering change within the exercise class

### Importance of physiotherapist supervision

The encouraging support of a specialist myeloma physiotherapist within supervised sessions appeared to be largely influential in shaping all participants’ experiences. A clear perceived impact of the physiotherapists was in easing safety concerns by instilling confidence to exercise and adjusting exercises to suit participants’ abilities. Participants suggested accountability to the physiotherapist motivated them to adhere to scheduled exercise sessions. Participants also indicated their accessibility to communication underpinned their positive relationship with the physiotherapists. Frequent and responsive communication may have furthered participants’ view of the physiotherapist as dependable whilst creating a collaborative relationship. However, one participant found the exercise intervention to be a difficult experience highlighting a lack of communication with physiotherapists about their concerns. Feeling unable to express their negative views about the intervention may have compounded their experience.

### Tailored exercise

Most of the interviewees reported that the supervised exercise regimen had a good variety of exercises, the right amount of contact (once per week), and duration (3 months). They felt it was enough time to build confidence they were doing the exercises correctly and long enough to begin establishing a routine. The exercises were set according to each participant’s ability, which meant that they found them hard yet achievable and were pleased to see improvements through progressions. This tailored approach resulted in them feeling valued and renewed their confidence and self-esteem. Taking part in the exercise intervention demonstrated to them that their physical body was able to do more than they had perceived. This challenged and broke down self-perceptions formed during treatment and gave them a renewed sense of hope.

### Limited engagement with behaviour change techniques

Behaviour change techniques were used to bring about a change in exercise habits. Some interviewees could see the benefit of the techniques to motivate change. However, others did not find them useful. This perceived lack of benefit was generated from the belief that techniques either were not right for them or they did not know how to use them. Participants engaged with the exercise logbook as it helped them adhere to the exercises, provided a visual reminder of their progression from the start, and helped them to set appropriate goals.

## Theme 4: Social support for exercise

### Social networks

Social support was received by interviewees from both family and peers, and was key to facilitating both participation in the programme and maintaining exercise at home. This was mostly offered verbally, with some family members actually participating in the home-based exercises with the participant, which was highlighted as motivating. It also gave patients the confidence to show family members what they had achieved in class.

### Group exercise sessions

The group exercise sessions were described as a source of social support, and a good opportunity to talk to other myeloma patients about their cancer journey and make comparisons with themselves in terms of recovery. Some found it important to be around myeloma survivors who were doing well and thought it was encouraging to see them perform strenuous exercises, despite having had extensive surgery. This gave them hope that if that happened to them, then they could still recover and regain their usual pattern of life.

Participants found being in a group made the exercise class enjoyable and were more likely to continue to take part. Interviewees reported a good rapport between themselves and the physiotherapists, which made the class good fun, go quickly, and led to friendships.

One participant reported a general dislike for support groups, believing that they tend to focus on the negatives. However, in contrast, they found this environment positive, as people were doing something meaningful to achieve their goals and improve themselves rather than focusing on what they could no longer do. Other participants reported not benefiting from being in a group with other survivors, as they identified themselves as introverted and did not enjoy sharing experiences.

## Theme 5: Maintaining things going forward

### Unsupervised exercise was challenging

A common difference between exercising with and without supervision among most of the participants was a reduced intensity during unsupervised home-based sessions. Not receiving the level of encouragement received from the physiotherapist was suggested to be a cause of reduced exercise intensity. Participants reported becoming dependent on the encouragement of the physiotherapist to evoke the effort to exercise intensely. In addition to the challenge of maintaining the exercise intensity in unsupervised sessions, participants shared how a lack of motivation and equipment posed a challenge to completing these sessions.

### Mechanisms to continue exercise

By the end of the exercise intervention, patients were aware of the benefits of exercise. They wanted to maintain their exercise levels going forward and the intervention had given them the confidence to do so. Participants reported developing new habits; adopting a variety of exercises into their lifestyle, until they had become routine. Some had adapted their exercise programme to ensure that it was manageable, achievable, and therefore maintainable.

Some had concerns about maintaining their current exercise levels when the programme ended, as it would require a lot of self-discipline. They felt that the programme had been quite intense and that this level of intensity would be difficult to maintain without the peer support of the group. Whilst they felt that social support from their friends and family would be crucial to achieving this, interviewees reported that they would have liked to have a drop-in exercise session, or the option to speak to someone, once the intervention had ended.

## Theme 6: Physical and mental benefits

### Improved physical health

Participants reported that taking part in the exercise intervention improved their functional status—physical fitness, energy, and strength. Some reported they were now physically able to do things they could not have done prior to their diagnosis.

### Improved self-efficacy

Participants’ felt that taking part had improved their self-efficacy, given them a sense of achievement, and enabled them to feel good about themselves for the first time since diagnosis.

Interviewees mentioned how they enjoyed being treated as a ‘normal’ person when receiving exercise instructions, and how the intervention helped them participate in ‘normal’ behaviours, which enabled them to feel freed of the perceived constraints of being a MM survivor.

Interviewees described how the intervention enabled them to have a more active lifestyle, and made them more aware of their own general health particularly that their body was not as weak as they had originally perceived. They discussed how they would have liked to have received the intervention earlier in their treatment**.**

## Discussion

To our knowledge, this is the first research study to qualitatively explore MM patients’ experiences of participating in a supervised exercise intervention. The main driver of participation in the study was altruistic but some saw it as a unique opportunity to guide them through recovery from the effects of treatment. Interviewees felt that exercise advice is lacking in current cancer treatment. The exercise intervention and particularly the physiotherapist support helped enhance patients’ self-efficacy for exercise. They derived benefits from having a MM physiotherapist, which increased adherence and helped participants feel safe. Group sessions offered informal support and friendship. Interviewees found the unsupervised sessions more challenging and had concerns about maintaining the intensity of their exercises once the programme had finished. The intervention was overwhelmingly considered a positive experience and participants stated they would recommend it to other MM patients.

One of the principal reported benefits of the class was improved physical function, which led to improvements in all aspects of the interviewees’ lives. In congruence with other studies, the participants we interviewed reported pain, fatigue, and reduced fitness which left them unable to return to work and perform activities of daily living thus relying on family members which left them feeling inadequate [4, 12]. Through exercise, they regained strength and fitness and felt able to regain their independence from their informal carers, and participate in activities they had not imagined they would be able to do again. This made them feel good about themselves and facilitated them to have the confidence to push their bodies and regain a sense of normality. Whilst improvements in QoL and physical functioning are seen in many exercise studies [5, 15] the MASCOT study found exercise had no quantitative effect on QoL [10]. However, these qualitative findings suggest the intervention had a profound impact on our interviewees, which was not captured by the quantitative measures used. QoL was captured in MASCOT using the FACT G. This QoL measure is typically used in cancer patients receiving therapy and so may not be as relevant to patients not currently receiving treatment. For example, items assessing acute symptoms i.e. “I have nausea” are typically no longer relevant to the same extent after treatment.

The participants we interviewed recognised they had reduced strength and fitness as a consequence of their disease. However, they had not received any advice from health professionals and instead were told to be careful of the fragility of their bones. This led them to avoid activity due to a fear of injury, further reinforced by family members. This highlights the need for health professionals to consider their language and its influence on how MM patients perceive their disease and the subsequent decisions they make. Hardcastle et al. [16] surveyed 123 international oncologists and found less than half provide PA advice with only 37% quoting the correct recommended guidelines. The main barriers oncologists reported were lack of clinic time, limited access to exercise specialists, and referral pathways. However, a qualitative paper interviewing 15 relapsed MM patients commented that clinicians need to be proactive in asking patients about their self-management strategies because as supported by a meta-aggregation of 11 qualitative studies, MM patients will not discuss their symptoms due to perceived time constraints during consultations [17]. Participants in our study indicated that input from a MM physiotherapist was influential in supporting them to build exercise confidence and develop self-management strategies. This demonstrates the potential value of utilising the knowledge and skills of other professions within myeloma medical care.

Indeed, our interviewees placed a significant value on having a MM physiotherapist deliver the exercise intervention. This is supported by the qualitative findings by Craike et al. [18] who found specialist MM clinicians were nominated by MM patients for supporting exercise because they can mitigate any safety concerns due to an understanding of the disease and treatment effects. Our interviewees found that this specialist knowledge enabled the physiotherapists to tailor the exercises to their abilities, which increased their confidence to exercise at a greater intensity and challenged their self-perceptions in a safe environment. This has also been observed in other cancer survivors [19].

Craike et al. [18] interviewed MM patients to establish their PA preferences, and reported a 50/50 split for supervised vs unsupervised sessions. The present study suggested our interviewees found exercising unsupervised more challenging because they relied on the encouragement from the physiotherapist and did not feel they were working as intensively at home because they did not have gym equipment, which reduced their motivation. One of the limitations of our main study was that 71% of MM patients declined the intervention due to travel; therefore, our sample may have been biased towards those who preferred supervised sessions [10]. However, studies with other groups of cancer patients suggest that supervised exercise can lead to higher intensity workouts and therefore greater effects for cancer patients in comparison to unsupervised [5, 20]. The present study’s findings demonstrate the importance of the support physiotherapists provided to MM patients within an exercise intervention and highlight some limitations around offering a home exercise programme. With nearly three-quarters of participants declining the intervention due to travel, future interventions should be designed to eliminate this barrier as far as possible and still maintain the intensity of face-to-face exercise sessions.

Additional benefits of supervised sessions are exercising within a group. MM patients want to talk to other myeloma survivors but do not get the opportunity [21]. Our interviewees were able to discuss their cancer journey and see how others were affected. There was good ‘banter’ between each other and the physiotherapist, these elements made the classes enjoyable, and friendships grew. Studies of cancer exercise rehabilitation interventions have also demonstrated group-based classes to be a source of invaluable support [19, 22]. However, within our interviews, we identified two participants who were apprehensive about group classes. One participant remained opposed to group classes preferring to keep their myeloma experiences private. However, the other participant changed their opinion, and found the exercise environment to be more positive than their past experiences of myeloma groups. Therefore, patients should be informed about the potential benefits of group exercise but providers should appreciate there is no ‘one size fits all’ approach, and offering both group and individual monitoring where possible will ensure inclusivity.

A concern often raised in intervention studies is maintenance, and our participants reported they wanted ongoing support, for example via a drop-in clinic. Behaviour change techniques have been found to support physical activity maintenance; however, our participants did not find these useful. This may be because over half of our interviewees were already exercisers (Table 3) but may also reflect the way the information was delivered. Participants did find the logbooks helped with self-monitoring and goal setting which have been identified as important aspects of successful maintenance of physical activity [23].

## Study limitations

Our findings reflect the experiences of those who chose to participate in the programme. However, further insights may have been gained from interviewing participants who did not engage with the programme or withdrew. Secondly, we only interviewed patients once they had finished the exercise intervention at 6 months; if we had interviewed them at 1 year, we may have had a better understanding of maintenance, long-term benefit gains, and what behaviour change techniques were useful.

Our sample for the interviews was mainly White (90%), male (70%), and functionally unaffected by MM with an ECOG score of 0 (85%). Within the intervention group who completed the MASCOT study, 78% were White, 51% were male, and 76% had an ECOG score of 0. Furthermore, recommended exercise guidelines were being met by 55% of participants prior to the intervention and the median age of participants was 64, whilst 67% of MM diagnoses occur above the age of 65 [24].

Convenience sampling was used to recruit participants. Therefore, our findings are not necessarily transferable to the wider MM population where individuals are generally older, have a higher proportion of African descent, and may have lower exercise levels and more co-morbidities resulting in functional impairment. Due to a large number of participants in ethnic minorities either not completing the intervention or declining to be interviewed, our results mainly reflect the views of White males.

We would therefore recommend future studies undertake patient and public work prior to the study starting to engage with minority ethnic communities about what tailored approaches might be used to address any barriers to participation [25], and use purposive recruitment to ensure a wider range of views and experiences are captured.

We would therefore recommend future studies undertake patient and public work prior to the study starting to engage with minority ethnic communities about what tailored approaches might be used to address any barriers of participation [25], and use purposive recruitment to ensure a wider range of views and experiences are captured.

## Clinical implications

The study’s findings support previous research-based recommendations, for the incorporation of exercise support within MM survivorship, but also emphasise the importance of physiotherapist supervision [26]. However, given limited resources within healthcare, supervised exercise interventions may not be widely feasible. Therefore, effective aspects of the intervention could be incorporated into care pathways. Providing a logbook that allows people to record exercise was highlighted as fundamental for adherence and could be cost-efficient. Providing training equipment at treatment centres could help MM patients address a barrier to exercising.

Similarly, transferring the programme to community settings could be explored. The COVID-19 pandemic has redefined accessibility to video technology in health care, and the provision of remote supervision via the many video platforms available could provide alternative methods to care [19].

## Conclusion

There is a lack of exercise support within MM care and patients’ experience reduced activity and self-efficacy. Our interviews of patients completing the MASCOT study highlight that exercise can improve MM patients’ physical function, aid their mental wellbeing, and support them to regain a sense of normality. Where feasible, successful rehabilitation programmes should include supervision and group sessions and be led by expert staff to ease safety concerns and increase engagement.

## Supplementary Information

Below is the link to the electronic supplementary material.

Supplementary file1 (DOCX 28 kb)

## Data Availability

Available upon request.

## Code availability

Available upon request.

## References

[1] Smittenaar CR, Petersen KA, Stewart K, Moitt N (2016). Cancer incidence and mortality projections in the UK until 2035. Br J Cancer.

[2] UK CR. Myeloma incidence by Sex and UK country 2019 [Available from: https://www.cancerresearchuk.org/health-professional/cancer-statistics/statistics-by-cancer-type/myeloma/incidence#heading-Zero.

[3] Statistics OfN. Cancer survival by stage at diagnosis for England 2019 [Available from: https://www.ons.gov.uk/peoplepopulationandcommunity/healthandsocialcare/conditionsanddiseases/datasets/cancersurvivalratescancersurvivalinenglandadultsdiagnosed.

[4] Ramsenthaler C, Osborne TR, Gao W, Siegert RJ, Edmonds PM, Schey SA (2016). The impact of disease-related symptoms and palliative care concerns on health-related quality of life in multiple myeloma: a multi-centre study. BMC Cancer.

[5] Stout NL, Baima J, Swisher AK, Winters-Stone KM, Welsh J (2017) A systematic review of exercise systematic reviews in the cancer literature (2005–2017). PM R 9(9S2):S347-S84 10.1016/j.pmrj.2017.07.074 PMC5679711 28942909

[6] Knips L, Bergenthal N, Streckmann F, Monsef I, Elter T, Skoetz N (2019) Aerobic physical exercise for adult patients with haematological malignancies. Cochrane Database Syst Rev 1:CD009075 10.1002/14651858.CD009075.pub3 PMC6354325 30702150

[7] Groeneveldt L, Mein G, Garrod R, Jewell AP, Van Someren K, Stephens R (2013). A mixed exercise training programme is feasible and safe and may improve quality of life and muscle strength in multiple myeloma survivors. BMC Cancer.

[8] Coleman EA, Coon S, Hall-Barrow J, Richards K, Gaylor D, Stewart B (2003). Feasibility of exercise during treatment for multiple myeloma. Cancer Nurs.

[9] Coleman EA, Goodwin JA, Kennedy R, Coon SK, Richards K, Enderlin C (2012). Effects of exercise on fatigue, sleep, and performance: a randomized trial. Oncol Nurs Forum.

[10] Koutoukidis DA, Land J, Hackshaw A, Heinrich M, McCourt O, Beeken RJ (2020). Fatigue, quality of life and physical fitness following an exercise intervention in multiple myeloma survivors (MASCOT): an exploratory randomised Phase 2 trial utilising a modified Zelen design. Br J Cancer.

[11] Coon SK, Coleman EA (2004). Keep moving: patients with myeloma talk about exercise and fatigue. Oncol Nurs Forum.

[12] Coon SK, Coleman EA (2004). Exercise decisions within the context of multiple myeloma, transplant, and fatigue. Cancer Nurs.

[13] Wood W, Runger D (2016). Psychology of habit. Annu Rev Psychol.

[14] Braun VaC, V (2006) Using thematic analysis in psychology. Qualitative Res Psychol

[15] Sweegers MG, Altenburg TM, Chinapaw MJ, Kalter J, Verdonck-de Leeuw IM, Courneya KS (2018). Which exercise prescriptions improve quality of life and physical function in patients with cancer during and following treatment? A systematic review and meta-analysis of randomised controlled trials. Br J Sports Med.

[16] Hardcastle SJ, Kane R, Chivers P, Hince D, Dean A, Higgs D (2018). Knowledge, attitudes, and practice of oncologists and oncology health care providers in promoting physical activity to cancer survivors: an international survey. Support Care Cancer.

[17] Hauksdottir B, Klinke ME, Gunnarsdottir S, Bjornsdottir K (2017). Patients' experiences with multiple myeloma: a meta-aggregation of qualitative studies. Oncol Nurs Forum.

[18] Craike M, Hose K, Courneya KS, Harrison SJ, Livingston PM (2017). Physical activity preferences for people living with multiple myeloma: a qualitative study. Cancer Nurs.

[19] Dennett AM, Peiris CL, Taylor NF, Reed MS, Shields N (2019). 'A good stepping stone to normality': a qualitative study of cancer survivors' experiences of an exercise-based rehabilitation program. Support Care Cancer.

[20] Baguley BJ, Bolam KA, Wright ORL, Skinner TL (2017) The effect of nutrition therapy and exercise on cancer-related fatigue and quality of life in men with prostate cancer: a systematic review. Nutrients 9(9) 10.3390/nu9091003 PMC5622763 28895922

[21] Kelly M, Dowling M (2011). Patients' lived experience of myeloma. Nurs Stand.

[22] Bennett AE, O'Neill L, Connolly D, Guinan EM, Boland L, Doyle SL (2018). Patient experiences of a physiotherapy-led multidisciplinary rehabilitative intervention after successful treatment for oesophago-gastric cancer. Support Care Cancer.

[23] Grimmett C, Corbett T, Brunet J, Shepherd J, Pinto BM, May CR (2019). Systematic review and meta-analysis of maintenance of physical activity behaviour change in cancer survivors. Int J Behav Nutr Phys Act.

[24] Kazandjian D (2016). Multiple myeloma epidemiology and survival: a unique malignancy. Semin Oncol.

[25] Redwood S, Gill PS (2013). Under-representation of minority ethnic groups in research–call for action. Br J Gen Pract.

[26] Walpole G, Clark H, Dowling M (2018). Myeloma patients' experiences of haematopoietic stem cell transplant: a qualitative thematic synthesis. Eur J Oncol Nurs.

