# Supplementary material for: Myeloma patients’ experiences of a supervised physical activity programme: a qualitative study
Source: Support Care Cancer. 2022 Apr 25;30(7):6273–86. doi: 10.1007/s00520-022-07062-x (PMC9035778; doi:10.1007/s00520-022-07062-x)
Supplement: Supplementary file 1 — Supplementary file1 (DOCX 28 kb) [file 520_2022_7062_MOESM1_ESM.docx]

**Supplementary Information**

Behavioral support during the exercise intervention

The behavioral change techniques included pros and cons, instructions on how to perform the behavior, behavioral

practice/rehearsal, demonstration of behavior, feedback on behavior, social reward, habit formation, behavioral

self-monitoring, behavioral goal setting with the SMART (specific, measurable, achievable, relevant, and timespecific) principle, action planning, feedback on behavior, mental rehearsal of successful performance, problem

solving, restructuring the physical environment, adding objects to the environment, and social support

(unspecified). Stress management techniques were also taught during the eighth session.

**Table 2: Interview Topic Guide**

**Myeloma study**

***Introduction***

*Introduce yourself and explain purpose of the interview: to explore the participant’s experience of the myeloma intervention and their experience of being a participant in the trial.  Ask permission to record the conversation. Assure confidentiality and ask participant to be as open and honest as they can be.*

| **Motivational factors** | - What was your main reason for taking part in this research? |
| --- | --- |
|  | *Probe:* to improve wellbeing or fitness/to help contribute to research/to receive additional monitoring by Health Professionals? |
| **Demotivating factors** | - Was there anything that made you think twice about taking part in the research? |
|  | *Probe*: personal concerns - practicalities/factors relating to your illness?  trial concerns - factors relating to the research itself? |
| **General trial feedback** | - How did you feel about being asked to take part in the research initially, i.e. before you were aware of the exercise intervention? |
|  | - How did you feel when approached about the exercise intervention element of the study? |
|  | *Prompt:* pleased/confused/surprised/annoyed/excited |
|  | - What did you expect to happen when you first enrolled on the study? |
|  | *Probe:* was it clear what being a participant would involve?  *Probe:* what about before being allocated to the exercise intervention and then after being allocated to the intervention? |
|  | - Do you feel you received enough on-going support from the Research team throughout the trial? |
|  | *Probe:* If no, what else do you think we could have done to provide more support? |
| **Assessments** | - How were the assessments? |
|  | - How far did you have to travel? Was this ok? |
|  | *Probe:* any problems with public transport/getting lifts? |
|  | - How did you find the length of assessments? |
|  | - What did you think of the questionnaires? |
|  | - Were you happy with the tests and measures that were carried out (e.g. testing breathing strength, muscle strength, wearing the accelerometer?) |
|  | *Probe:* Was there anything you felt should have been done differently?  Probe: Was there anything you were uncomfortable with? |
|  | - How did you perceive the skills and knowledge of the Practitioner/s who saw you for your baseline and follow-up assessments? |
|  | *Probe:* did you like their approach?  *Probe:* how was their manner? |
| **The exercise intervention** | - Was the travel to your gym sessions ok? |
|  | - How did you feel about the frequency of gym sessions? |
|  | *Prompt*: was it useful to attend the gym as often as you did? Or were the session too frequent for your liking?  if so how often would you have liked to have them? |
|  | - How did you feel about the level of contact you had with the Physio? |
|  | *Prompt:* would you have liked any more or less contact?  If so why? |
|  | - How did you perceive the skills and knowledge of the Physiotherapist/s? |
|  | *Probe:* how did you find their approach?  *Probe:* how was their manner? |
|  | - What did you think of the exercises you were prescribed? |
|  | *Probe:* did you feel they were either too arduous, or not arduous enough?  *Probe:* did you have any concerns or worries? |
|  | - Were you able to carry out the prescribed exercises ok in the gym? |
|  | - How about at home? |
|  | - When you weren’t able to carry out the exercises why was this? |
|  | - How did doing the exercise make you feel directly afterwards? |
|  | - How did doing the exercise make you feel on the whole? |
|  | *Prompt:* enjoyable/happy/energised/tired/increased levels of pain |
|  | - How did you find being in a group with other Myeloma patients, when in the gym? |
|  | *Prompt:* did it result in extra support/reduce contact time with the Physio? |
| **Behaviour change support** | - What did you think of the behaviour change support provided? |
|  | - Were the techniques new to you or had you heard about any of them before? |
|  | *Probe:* if so have you used any of them before? |
|  | - How useful would you say the behaviour change support was in terms of helping you change your behaviours? |
|  | *Probe:* which elements were most helpful. E.g. log book, goal setting, rewards?  *Probe:* which were least helpful? |
|  | - What did you think of the log book? |
|  | *Probe:* did you fill it out?  *Probe:* did you use it to reflect on how you’d done over the weeks?  *Probe:* was there anything about it you would change? |
| **General** | - Can you tell me how you felt, overall, about being a participant in this research? |
|  | *Probe:* what were the benefits of taking part?  *Probe:* What were the disadvantages? |
|  | - Did you have support from family and or friends? |
|  | *Prompt:* was the support practical, emotional or both?  *Probe:* if yes, how important was that to you?  if no, do you think this could have helped? |
|  | - Would you recommend taking part to other people? |
|  | - Do you think that you have learned anything as a result of taking part in the trial? |
|  | *Probe:* do you think you have gained anything? |
|  | - Is there anything you think we should have done differently? |
|  | - Do you think you will continue with the exercises when the trial is over? |
|  | *Probe:* If no, what sort of things might prevent you from carrying on? |
|  | - Is there anything else you would like to say about the trial, that we’ve not already talked about? |

***Concluding comments and thanks***

*Thank the participant for their time today and ongoing and let them know where they can contact you in the future if they do have any additional comments.*
